# Efficacy of intravenous immunoglobulin (IVIg) on COVID-19-related neurological disorders over the last 2 years: an up-to-date narrative review

**DOI:** 10.3389/fnins.2023.1159929

**Published:** 2023-04-25

**Authors:** Paolo Manganotti, Gabriele Garascia, Giovanni Furlanis, Alex Buoite Stella

**Affiliations:** Department of Medicine, Surgery and Health Sciences, University Hospital of Trieste, Clinical Unit of Neurology, Azienda Sanitaria Universitaria Giuliano Isontina (ASUGI), University of Trieste, Trieste, Italy

**Keywords:** SARS-CoV-2, COVID-19, intravenous immunoglobulin (IVIG) administration, inflammation, immunoglobulins

## Abstract

**Introduction:**

Among the clinical manifestations of SARS-CoV-2 infection, neurological features have been commonly reported and the state-of-the-art technique suggests several mechanisms of action providing a pathophysiological rationale for central and peripheral neurological system involvement. However, during the 1^st^ months of the pandemic, clinicians were challenged to find the best therapeutic options to treat COVID-19-related neurological conditions.

**Methods:**

We explored the indexed medical literature in order to answer the question of whether IVIg could be included as a valid weapon in the therapeutic arsenal against COVID-19-induced neurological disorders.

**Results:**

Virtually, all reviewed studies were in agreement of detecting an acceptable to great efficacy upon IVIg employment in neurological diseases, with no or mild adverse effects. In the first part of this narrative review, the interaction of SARS-CoV-2 with the nervous system has been discussed and the IVIg mechanisms of action were reviewed. In the second part, we collected scientific literature data over the last 2 years to discuss the use of IVIg therapy in different neuro-COVID conditions, thus providing a summary of the treatment strategies and key findings.

**Discussion:**

Intravenous immunoglobulin (IVIg) therapy is a versatile tool with multiple molecular targets and mechanisms of action that might respond to some of the suggested effects of infection through inflammatory and autoimmune responses. As such, IVIg therapy has been used in several COVID-19-related neurological diseases, including polyneuropathies, encephalitis, and status epilepticus, and results have often shown improvement of symptoms, thus suggesting IVIg treatment to be safe and effective.

## 1. Introduction

The viral agent SARS-CoV-2 is responsible for COronaVIrus Disease-19 (COVID-19), a syndrome in which respiratory symptoms play the main role. Nevertheless, since the beginning of the pandemic in late 2020, central and peripheral neurological diseases associated with the infection were reported, leading not only to impactful and early symptoms such as stroke, encephalitis, epilepsy, myelitis, and inflammatory polyneuropathy but also milder and long-lasting sequelae as drowsiness, loss of memory, brain fog and headache. These symptoms have been reported in a high proportion of patients, and the term “neuro-COVID” has been coined (Leonardi et al., 2020). Despite a possible neuroinvasive ability of SARS-CoV-2, current scientific evidence suggests an inflammatory storm subsequent to the immune system activation induced by SARS-CoV-2 as the principal mechanism behind the nervous system injury (Thepmankorn et al., 2021).

Intravenous immunoglobulin (IVIg) is a consolidated therapeutic tool for the treatment of some systemic inflammation-based neurological diseases (Hughes et al., 2009). Based on this clinical experience, the possibility to treat COVID-19-related dysimmune neurological diseases, especially at the beginning of the pandemic, has been proposed as a potential life-saving therapeutic option for several patients, despite the few information known at the time about the virus–host interaction (Gharebaghi et al., 2020). The use of IVIg in COVID-19 and its neurological complications have increased with a dramatic clinical impact on available treatments as well as providing new insights on this pharmacological therapy and autoimmune consequences of infection (Danieli et al., 2021). In this narrative review, we report and discuss the data from 130 reported series on the Neurology and Neuropsychiatry of COVID-19, providing a comprehensive overview of patients with neurological immune-mediated complications of SARS-CoV-2, preferentially treated with immunoglobulins as a single therapy for their neurological disease.

### 1.1. Intravenous immunoglobulins

IVIg composition consists of human antibodies isolated and concentrated from healthy donors. The amount of immunoglobulin G (IgG) is markedly predominant (approximately 95%), whereas IgA and IgM are present in trace (Liu X. et al., 2020). A pool of at least 1,000 donors is the source of these plasmatic products. Adequate testing for the Human Immunodeficiency Virus (HIV) 1 and 2, hepatotropic viruses as well as for several other infectious agents must precede the medical use. High titers of ABO antibodies are also to be excluded in order to avoid hemolytic reactions. Due to the robust measures put in place to screen donors and inactivate possible pathogens, the safety of immunoglobulin formulations has been excellent for many years (Quinti et al., 2002). The requirements of the final product are at least 90% intact IgG and similar proportions to the human plasma for the other subclasses of antibodies (Lünemann et al., 2016).

The basic structure of an IgG molecule is made up of polypeptide chains and consists of two identical heavy chains and two identical light chains forming a y-shaped structure. Two antigen-binding Fragments (Fab) and one crystallizable Fragment (Fc) are the results of enzymatic digestion by papain protease. Whereas IgG-Fab mediates highly specific interactions with the antigen by selectively targeting a molecular region called the epitope, the crystallizable region IgG-Fc is able to bind to Fc-gamma-receptors (FcγRs) on the membrane of immune cells obtaining various immunoregulatory effects and even to complement proteins, leading to their inactivation (Arumugham and Rayi, 2022).

IVIg can be usually administered at different dosages because of their desired therapeutic action. Thus, low and moderate quantities function as a substitutional therapy for primary or acquired immunodeficiencies, while high doses constitute immunomodulatory therapy for autoimmune and inflammatory diseases (Kerr et al., 2014).

### 1.2. IVIg mechanisms of action

It has been highlighted that an important target of some IVIg antibodies is a motif expressed on a variety of cell surfaces and adhesion proteins, specifically a 10-peptide sequence containing the aminoacidic triad Arginine–Glycine–Aspartate (RGD) (Vassilev et al., 1999). Some experiences have shown a dramatic switch from proinflammatory to anti-inflammatory conditions, a result due to the F(ab)′2-mediated neutralization of inflammatory cytokines, chemokines, and complement molecules, along with the modulation of immune cell apoptosis (Liu X. et al., 2020). Therefore, by specific antibodies, IVIg might interfere with the adhesion between B cells and fibronectin and with platelet aggregation (Vassilev et al., 1999), as well as with B cell development, by directly neutralizing B-cell growth factors (Ballow, 2011). Both B-cell receptor–dependent and independent antigen presentation have been inhibited in purified murine B cells (Paquin Proulx et al., 2010). Nevertheless, IVIg seems to be active also on T-cells. In experimental autoimmune encephalomyelitis, a murine model of multiple sclerosis, animals treated with IVIg experienced an expansion of their population of CD4, CD25, and FOXP3-positive regulatory T cells, resulting in a diminished number of autoimmune events. This activating effect seems ascribable to specific regions of the Fc fragment (De Groot et al., 2008; Ephrem et al., 2008).

Other Fc-mediated mechanisms involve saturation of activating FcγRs and FcRn and upregulation of inhibitory FcγRIIB. These effects have been emphasized by high-dose IVIg therapy (Liu X. et al., 2020). The neonatal Fc receptor (FcRn) is a specialized receptor capable of sparing IgG from lysosome-mediated degradation and returning the IgG intact to plasma circulation. The long half-life of 21 to 25 days of serum IgG is a major product of these receptors (Junghans and Anderson, 1996). In antibody-mediated autoimmune diseases, enhanced catabolism of the autoantibodies through IVIg-related saturation of the FcRn receptor found in the endocytic vesicles of endothelial cells has been described (Yu and Lennon, 1999). Besides, IVIg can stimulate inhibitory FcgRIIb receptors. They have been found on a variety of cell types (including macrophages, B cells, and a subpopulation of T cells) and appear to provide these cellular types an inhibitory signal triggered by an immunoregulatory tyrosine-based inhibition motif or ITIM (Daëron, 1997). Another mechanism by which IVIg can inhibit B-cell proliferation is direct binding and neutralization of B-cell trophic factors such as BAFF and APRIL proteins (Zhu et al., 2006).

Immature dendritic cells secrete increased levels of anti-inflammatory cytokines and decreased levels of proinflammatory cytokines in coincidence with a high-dose IVIg treatment (Bayry et al., 2003). This also led to a reduction in the number of NK cells and their cytotoxic activity in patients with autoimmune diseases (Papaserafeim et al., 2020). If on a side IVIg therapy could reduce the production of cytokines such as interLeuchin-1 (IL-1), tumor necrosis factor (TNF), and interferon-γ induced by bacterial superantigens or lipopolysaccharide in peripheral blood mononuclear cells, on the other side, it may increase the production of IL-1 receptor antagonist, a decoy receptor that counteracts IL-1 signaling (Gupta et al., 2001).

The immunomodulatory effects of IVIg are further imputable to F(ab)′ 2-mediated neutralization of the C3a and C5a factors of complement and a dose-dependent interaction between the Fc fragment and complement C1q, C3b, and C4 factors (Lutz et al., 2004). IVIg can also reduce the activation of the C3 component and the formation of the membranolytic attack complex, the main effector of complement-induced cytolysis (Dalakas, 2019).

Another possible way by which IVIg seems to modify the autoimmune disease processes is an idiotype/anti-idiotype interaction. Idiotypes are antigenic determinants of immunoglobulins that can be targeted in their turn by other antibodies with an immunomodulatory function (Abu-Shakra and Shoenfeld, 2007). This could contribute to remyelination in patients with Guillain-Barré Syndrome (GBS) and chronic inflammatory demyelinating polyneuropathy (Malik et al., 1996). Anti-idiotypic antibodies might also act on the FcgRIIb receptors on B cells to generate a stop signal to the B cells synthesizing autoimmune antibodies (Gergely and Sármay, 1996).

A last hypothesized mechanism of action of IVIg, in the specific case of SARS-CoV-2, is the direct targeting and neutralization of the virus, by cross-reacting antibodies naturally present in donors' plasma due to previous infections with other related coronaviruses, including those of the common cold (Díez et al., 2020; Dalakas et al., 2021).

As further evidence of the fact that IVIg is not tout court immunosuppressive but immunomodulatory, different clinical studies have shown their therapeutic effect on encephalitis secondary to *Mycoplasma pneumoniae* infection in children (Sakoulas, 2001; Daba et al., 2019).

## 2. The neurotropism of SARS-CoV-2

The tropism of human Coronaviruses for the nervous system has already been demonstrated by several studies, *in vitro* on primary cultures of human astrocyte, microglia, and neuronal cells (Arbour et al., 1999) *in vivo* on animal models (Jacomy and Talbot, 2003), and even in autopsies using *in situ* hybridization of the cortical and hypothalamic tissues (Gu et al., 2005).

The novel pandemic agent SARS-CoV-2 uses as access key its transmembrane spike (S) glycoprotein that forms homotrimers protruding from the pathogen surface. Molecular interactions between the angiotensin-converting enzyme 2 (ACE2) and the S protein, through which SARS-CoV-2 can enter cells, have been well elucidated (Walls et al., 2020). The ACE2 receptor is expressed on the cell surface of multiple organs, especially in the alveolar epithelial type II cells in the lungs, but also in the brain. In the past, receptorial distribution in the central nervous system (CNS) was considered exclusively vascular (Hamming et al., 2004). To date, it is of note that ACE2 is expressed in many excitatory and inhibitory neurons and in some astrocytes and oligodendrocytes as well (Chen et al., 2020).

Two main pathways for the invasion of the CNS have been described: the hematogenous one that is ultimately subdivided into direct and immune cell-mediated pathways; the neurogenic one postulates via both neuronal ACE-2 receptors and retrograde axonal transports (Keyhanian et al., 2020). The mechanism by which SARS-CoV-2 would directly invade the CNS through the hematic flow may be based on a previous brain–blood barrier disruption. The virus might cause direct cell stress and endothelial cell activation. By cascade, they would produce increased levels of proteases, leading to the degradation of tight junctions (Alquisiras-Burgos et al., 2021). Otherwise, thanks to a better binding to ACE2 due to the sluggish movement of the blood within the microcirculation, the viral particles could bud from the capillary endothelium, thus destroying it (Baig et al., 2020). The indirect hematogenous route involves the extravasation of immune cells, including macrophages and lymphocytes, into meninges and cerebrospinal fluid (CSF). They might also use the meningeal lymphatic system as a privileged way to the brain tissue. The last two ways constitute the so-called “Trojan horse” mechanism (Barrantes, 2020).

The neurogenic route consists of the viral ability to invade peripheral nerve terminals and circulate in the retrograde way along nerve synapses to the dorsal root ganglions (Zubair et al., 2020). Neuronal spreading has been indicated both via endocytic/exocytic vesicles sent from a nerve cell to others (Li et al., 2020) but conceivably it would also employ the fast axonal transport, realizable either in the antidromic or in the orthodromic direction (Dubé et al., 2018). The virus might also utilize the retrograde transport system from the gustatory receptor cells in the tongue to the neurons of the nucleus solitarius in the medulla, thereby causing ageusia (Vaira et al., 2020). Another hypothesized neurogenic pathway seemed to directly involve the olfactory nerve, as formerly observed in other coronavirus infections after intranasal inoculation (Li et al., 2016). Recently, due to the ascertained lack of the entry protein expression in olfactory neurons, this thesis was critically denied (Butowt et al., 2021).

## 3. The cytokine storm in neuro-COVID

In COVID-19 several immunochemical alterations have been identified, such as a durable decrease in lymphocyte number and increases in neutrophils, dimer-D, and certain cytokine and chemokine levels. For instance, IL-2, IL-6, IL-7, IL-10, TNF and leucocyte growth factors are commonly enhanced, especially in the most critical patients (Costela-Ruiz et al., 2020). SARS-CoV-2 might provoke an immune shift with an increase in the number of naïve and Th-17 T cells and a reduction in the number of memory and regulatory T cells leading to a proinflammatory status (Keyhanian et al., 2020). Within the CNS, microglia activation has been described, with a raise in reactive oxygen species (ROS) and cytokine levels. Oxidative stress, neuronal excitotoxicity, and dysfunction in synaptic pruning are possible consequences. There may be a “cross-talk” mediated by neuropeptides between the cytokine network of the CNS and that of the rest of the organism (Bhaskar et al., 2020).

For many years, IL-6 has been recognized as a hypercoagulability-linked factor, thus putting at risk of ischemic stroke (Stouthard et al., 1996). Upon COVID-19, this cytokine increases proportionally to the disease severity, accounting for a prognostic factor (Liu F. et al., 2020). Another molecule boosted in level and based on a general pro-inflammatory diathesis in the CNS is the monocyte chemoattractant protein-1. It is expressed by neurons, astrocytes, and microglia (Farhadian et al., 2020). In CSF, markedly increased levels of IL-6, 8, 10 and TNF can be found, although in the absence of the virus (Benameur et al., 2020). IL-8, particularly, may be used as a biomarker of an active autoimmune process against the CNS, since it can be found in CSF at an even higher level than in serum upon COVID-19-related GBS (Manganotti et al., 2020).

The cytokine storm could generate aggressive effector cells that are capable of harming the brain tissues. They would be the product of the activation of resident glial cells (Yachou et al., 2020). Vascular alterations induced by elevated inflammatory markers may result in the posterior reversible encephalopathy syndrome (Princiotta Cariddi et al., 2020). Besides, proinflammatory cytokines could focally destroy the blood–brain barrier, inducing edema and hence necrosis. This has also been correlated with the development of acute necrotizing encephalopathy (Virhammar et al., 2020).

Interestingly, a neuroinflammation-triggered Gamma-AminoButyric Acid (GABA)ergic system impairment, particularly at the central level, may lead to neuromotor fatigue, cognitive dysfunction, executive deficits, and mood modifications even in post-COVID-19 patients (Ortelli et al., 2021). Furthermore, the cytokine storm is the origin of a delayed headache for 7–10 days after the onset of the infection symptoms, accompanied by photophobia and neck stiffness (Belvis, 2020).

## 4. SARS-CoV-2-induced autoimmunity

COVID-19 has been associated with classical neurological autoimmune disorders, such as GBS spectrum, acute disseminated encephalomyelitis (ADEM), and encephalitis (both seronegative and seropositive). Though caution is required for an eventual causal attribution to specific antibody patterns, at least indirect autoimmune mechanisms can be postulated (Ariño et al., 2022).

SARS-CoV-2 can cause injury through autoimmune processes, as some epitopes from SARS-CoV-2 might induce a cross-reactivity with autoantigens (Liu et al., 2021). Some proteins expressed in neurons of the pre-Bötzinger complex in the human brainstem such as DAB1, AIFM, and SURF1 could be involved in molecular mimicry with antigens of SARS-CoV-2 and this may contribute to the respiratory failure in COVID-19 (Lucchese and Flöel, 2020a).

Another molecular-mimicry phenomenon might occur between viral proteins and human molecular chaperones, most of which act as heat shock proteins (Marino Gammazza et al., 2020). Indeed, heat shock proteins 90 and 60, associated with GBS and other immune-mediated polyneuropathies, in comparative analyses between viral aminoacidic chains and human autoantigens have been demonstrated to share the same sequence of some SARS-CoV-2 immunoreactive epitopes (Lucchese and Flöel, 2020b). It is possible that epitopes within the spike-bearing gangliosides trigger an immune cross-reaction against the sugar residues of surface peripheral nerve glycolipids (Uncini et al., 2020).

In a study by Guilmot et al. (2021), 3 of 15 patients with COVID-19-related neurological conditions presented with CSF pleocytosis and anti-GD1b IgG antibodies, accompanied by the involvement of multiple cranial nerves and cauda equina. A high frequency of CSF antineuronal and antiglial autoantibodies targeting neuropils, astrocytes, or medium-sized brain blood vessels have been reported in critically ill COVID-19 patients; more than one patient had IgG autoantibodies (Franke et al., 2021).

In patients neurologically ill due to SARS-CoV-2 infection, anatomic immunostaining can show intrathecal autoantibodies and hippocampal, olfactory, and cerebrovascular autoantibody deposition. Possible target autoantigens are THAP3 and IFT88. IFT88 is a ciliary protein whose mutation causes anosmia in mice, while THAP3 protein may be implicated in genetic causes of dystonia (Song et al., 2021).

## 5. COVID-19-associated neurological conditions treated with IVIg and indirect evidence of the autoimmune mechanisms of clinical complications

In the last years characterized by the COVID-19 pandemic, several studies, including case reports and case series, have described the neurological features of SARS-CoV-2 infection, and the efficacy and safety of IVIg treatment have been often reported (Table 1).

**Table 1:** Summary of studies related to IVIg treatment in COVID-19 patients presenting neurological conditions.

**References**	**Neurological disease**	**IVIg-treated**	**IVIg regimen**	**Key findings**	**Associated immunosuppressants^a, b^**
Sriwastava et al. (2021)	COVID-19-related Guillain-Barré syndrome.	44 patients. All patients of the study were 50: 33 in the AIDP group (mean age 62 ± 9.9 years), 17 in the non-AIDP group (mean age 52 ± 16.3).	0.4 g/kg/day for 14 AIDP and 8 non-AIDP patients. 2 g/kg for 6 AIDP and 2 non-AIDP patients. All over 5 days.	Either full or partial recovery in almost all patients. No data about side effects.	Four patients also received plasma exchange for GBS treatment; 1 also received tocilizumab for COVID-19 treatment.
Filosto et al. (2021)	COVID-19-related Guillain-Barré syndrome.	25 patients (mean age of IVIg + non IVIg- treated of 59.2 ± 12.1 years).	Mostly 0.4 g/kg/day for 5 days.	Improvement in at least the 80% of cases. No data about side effects.	No data about concomitant immunosuppressant treatments.
Abu-Rumeileh et al. (2021)	COVID-19-related Guillan-Barré syndrome spectrum.	60 patients (mean age of IVIg + non IVIg-treated of 55 ± 17 years).	Mostly 0.4 g/kg/day for 5 days.	Variable improvement of at least some symptoms in almost all patients. No data about side effects.	4 patients also received plasma exchange for GBS treatment; 6 also received corticosteroids for COVID-19 treatment; 2 received also tocilizumab for COVID-19 treatment.
Elzouki et al. (2021)	COVID-19-related Guillan-Barré syndrome spectrum.	85 patients treated only with IVIg.	Almost all patients treated with 0.4 g/kg/day for 5 days.	64 patients had a favorable outcome, 17 poor, 4 died. No data about side effects.	6 patients also received plasma exchange and 2 methylprednisolone for GBS treatments.
Garcia et al. (2021)	COVID-19-related Guillain-Barré syndrome during pregnancy.	A 22-year-old pregnant woman.	0.4 g/kg/day for 5 days.	Complete recovery within 1 month after IVIg cycle. No adverse events. Vaginal birth at term.	No plasma exchange neither immunosuppressant treatments other than IVIg.
Assini et al. (2020)	COVID-19-related Guillain-Barré syndrome spectrum.	Two men aged 55 and 60 years.	0.4 g/kg/day for 5 days.	Marked improvement. No adverse events.	No plasma exchange neither immunosuppressant treatments other than IVIg.
Khaja et al. (2020)	Bilateral facial palsy in COVID-19-related Guillain-Barré syndrome.	A 44-year-old man.	0.4 g/kg/day for 5 days.	Nearly complete recovery. No adverse events.	No plasma exchange neither immunosuppressant treatments other than IVIg.
Gutiérrez-Ortiz et al. (2020)	COVID-19-related Miller Fisher syndrome.	A 50-year-old man.	0.4 g/kg/day for 5 days.	Almost complete recovery. No adverse events.	No plasma exchange neither immunosuppressant treatments other than IVIg.
Reyes-Bueno et al. (2020)	COVID-19-related Miller Fisher syndrome.	A 51-year-old woman.	0.4 g/kg/day for 5 days.	Progressive improvement. No adverse events.	No plasma exchange neither immunosuppressant treatments other than IVIg.
Dinkin et al. (2020)	COVID-19-related Miller-Fisher syndrome.	A 36-year-old man.	2g/kg over three days	Partial recovery. No adverse events.	No plasma exchange neither immunosuppressant treatments other than IVIg.
Yousuf et al. (2021)	COVID-19-related autoimmune encephalitis.	A 60-year-old man.	0.4 g/kg/day for 5 days.	Almost complete recovery. No adverse events.	No data about concomitant immunosuppressant treatments.
Chenna et al. (2021)	COVID-19-related encephalitis.	A 58-year-old man.	0.4 g/kg/day for 5 days.	Complete recovery. No adverse events.	Methylprednisolone for COVID-19 treatment.
McAlpine et al. (2021)	COVID-19-related autoimmune encephalitis.	A 30-year-old man.	2 g/kg of IVIg over 3 days	Complete recovery. No adverse events.	No plasma exchange neither immunosuppressant treatments other than IVIg.
Burr et al. (2021)	COVID-19-related autoimmune encephalitis.	A 23-month-old female.	2g/kg (over a non-specified number of days).	Complete recovery. No adverse events.	Intravenous methylprednisolone 30 mg/kg/day for five days (ineffective).
Gaughan et al. (2021)	COVID-19-associated encephalitis.	A 16-year-old female.	0.4 g/kg/day for 5 days for one cycle. A second cycle was started and promptly discontinued, due to an adverse reaction.	Nearly complete recovery after 6 months. No adverse events in the first cycle. A widespread rash in the second cycle.	Intravenous methylprednisolone 1 g/day for 3 days after the first IVIg cycle.
Fukushima et al. (2021)	Post-infectious COVID-related encephalitis.	A 20-year-old man.	0.4 g/kg/day for 5 days.	Complete recovery within 2 months. Remission of MRI lesions. No major side effects.	No plasma exchange neither immunosuppressant treatments other than IVIg.
Delamarre et al. (2020)	COVID-19–associated acute necrotising encephalopathy.	A 51-year-old male.	Total dose of 2 g/kg over 5 days.	Significant improvement of MRI lesions after 35 days and complete motor recovery. No adverse events.	Intravenous methylprednisolone 1 g/day for 3 days concomitantly with IVIg.
El-Zein et al. (2020)	COVID-19-associated meningoencephalitis.	A 40-year-old male.	0.4 g/kg/day for 5 days.	Complete recovery 2 months after the discharge. No adverse events.	No plasma exchange neither immunosuppressant treatments other than IVIg.
Muccioli et al. (2021)	COVID-19-related encephalopathy.	Five patients (including two females) with a mean age of 66.8 years.	0.4 g/kg/day for a minimum of 3 and a maximum of 5 days.	Almost complete clinical and radiological recovery soon after the therapy. No adverse events.	2 patients also received tocilizumab and low-dose corticosteroids and 1 also received tocilizumab alone for COVID-19 treatment; 1 also received methylprednisolone 1 g/day for 5 days for encephalopathy treatment.
Abdel-Mannan et al. (2020)	COVID-19-related encephalopathy.	A 15-year-old female.	One dose of 1 g/kg.	Resolution. Discharge after 18 days. No adverse events.	No plasma exchange neither immunosuppressant treatments other than IVIg in this selected case.
Delorme et al. (2020)	COVID-19-related encephalopathy.	A 72-year-old man.	2 g/kg over a non-specified number of days.	Gradual recovery. No adverse events.	No plasma exchange neither immunosuppressant treatments other than IVIg in this selected case.
Manganotti et al. (2021b)	New-onset refractory status epilepticus (NORSE) in COVID-19.	Two males aged 37 and 71 years.	0.4 g/kg/day for 5 days for one in the first case and two cycles in the second.	Partial decrease of antiepileptic drugs without severe residual cognitive impairment. No adverse events.	No plasma exchange neither immunosuppressant treatments other than IVIg.
Leelamani and Mohammed Rajab (2021)	COVID-19-related NORSE.	A young adult female.	0.4 g/kg/day for 5 days.	No focal neurological deficits after 15 days. No adverse events.	No plasma exchange neither immunosuppressant treatments other than IVIg.
Stoian et al. (2021)	COVID-19-related critical illness polyneuropathy.	A 54-year-old woman.	0.4 g/kg/day for 5 days.	Marked improvement. No side effects.	No plasma exchange neither immunosuppressant treatments other than IVIg in this selected case.
Novak (2020)	Post-COVID-19 orthostatic hypoperfusion syndrome (OCHOS) and small fiber neuropathy (SFN).	A 64-year-old female.	2 g/kg/month for 2 months, decreased to 1 g/kg/month.	Marked improvement of all symptoms. IVIG-induced headaches.	No plasma exchange neither immunosuppressant treatments other than IVIg.
Saleh et al. (2021)	COVID-19-related polyneuropathy.	A 56-year-old female.	0.4 g/kg/day for 5 days. Another dose of 90 g after 4 weeks.	IVIg-induced headaches after the first cycle. None after the second.	No plasma exchange neither immunosuppressant treatments other than IVIg.
Manganotti et al. (2021a)	Guillain-Barré spectrum polyradiculoneuritis and cranial polyneuritis.	Four males aged 72, 72, 76, and 94 years and a 49-year-old female.	0.4 g/kg/day for 5 days.	Partial resolution of symptoms. No adverse events.	No plasma exchange neither immunosuppressant treatments other than IVIg in these selected cases.
Delly et al. (2020)	Myasthenic crisis in concomitant COVID-19 syndrome.	A 56-year-old female.	A first cycle of 0.4 g/kg/day for 5 days. A second cycle of 650 mg/kg for 2 days in a row.	Greatly improved ventilatory function and motility. Not reported side effects.	No plasma exchange neither immunosuppressant treatments other than IVIg.
Huber et al. (2020)	COVID-19-related onset of ocular myasthenia gravis.	A 21-year-old woman	0.4 g/kg/day for 5 days.	Regression of ocular deficits. No adverse events.	No plasma exchange neither immunosuppressant treatments other than IVIg.
Parsons et al. (2020)	COVID-19-associated acute disseminated encephalomyelitis.	A 51-year-old female.	0.4 g/kg/day for 5 days.	Improvement of the alertness. Not reported side effects.	Intravenous methylprednisolone 1 g/day for 5 days (ineffective) prior to IVIg.
Gupta et al. (2021)	COVID-19-related myositis.	A 49-year-old female.	0.4 g/kg/day for 5 days.	Complete recovery in a few weeks. No side effects.	No plasma exchange neither immunosuppressant treatments other than IVIg.
Ishaq et al. (2021)	COVID-19 related opsoclonus myoclonus syndrome.	A 63-year-old male.	2 g/kg in 5 divided doses.	Gradual complete recovery. No adverse events.	Intravenous methylprednisolone 1 g/day for 5 days (ineffective) prior to IVIg.

^a^Hydroxychloroquine (for COVID-19 treatment) has not been included. ^b^Unless otherwise specified, corticosteroids were given prior to IVIg in order to treat COVID-19 (low dosage) or the COVID-19-related neurological condition (high dosage).

### 5.1. Guillain-Barré syndrome spectrum

Abu-Rumeileh et al. (2021) systematically reviewed 73 COVID-19-related cases of the so-called “GBS spectrum”, a group of diseases that share some clinical presentation and pathogenetic mechanisms with the GBS. A majority of them, equal to 63%, were represented by males; no significant difference in age was found (mean age of IVIg + non-IVIg-treated of 55 ± 17 years). All reported GBS cases (*n* = 72) except two were symptomatic for COVID-19 with various severity. A total of 60 patients were treated with IVIg. The most used dosage was the standard 0.4 g/kg daily for 5 days. Almost all of them showed a variable improvement of at least some symptoms, while someone suffered remission. More exactly, out of 68 patients, 72.1% (49/68) demonstrated clinical improvement with partial or complete recovery, 10.3% (7/68) showed no improvement, 11.8% (8/68) still required critical care treatment, and 5.8% (4/68) died. Unfortunately, the authors did not distinguish the outcomes between patients treated with IVIg and those treated with other therapies. Furthermore, the aforementioned study did not focus on the possible side effects of IVIg therapy, and it seems plausible that they were not of great entity.

Elzouki et al. (2021) examined 64 patients (60%) diagnosed with acute inflammatory demyelinating polyneuropathy (AIDP), 18 (17.1%) with acute motor sensory axonal neuropathy (AMSAN), 11 (10.5%) with acute motor axonal neuropathy (AMAN), nine (8.6%) with Miller–Fisher Syndrome (MFS), and four (3.8%) with bifacial weakness with paraesthesia. In 12 patients (11%), the subtype of GBS was not specified. Eighty-five patients received only IVIg and no other treatments. Of them, almost all were treated with the classical dosage of 0.4 g/kg/day for 5 days; 64 had a favorable outcome, 17 poor, and 4 died. In this study, adverse events were not collected and reported. In the same study, Elzouki et al. (2021) described two clinical cases of, respectively, a 49-year-old male patient with AIDP and a 43-year-old male patient with AMSAN. Both these neurological disorders belong to the GBS spectrum. The first patient was admitted to the hospital with symptoms of an acute progressive symmetrical weakness ascending from the distal lower extremities that had gradually worsened before 5 days. Muscle examination showed weakness in the four limbs. Deep tendon reflexes were absent bilaterally with reduced sensitivity in the distal limbs. CSF analysis showed protein–cellular dissociation. These and the neurophysiological findings were consistent with an AIDP. The patient was treated with 0.4 g/kg/day of IVIg for 5 days. He showed a significant improvement in his strength with complete recovery after 6 weeks of follow up. The second patient presented with acute onset of progressive ascending weakness over the 7 days prior to admission. Paraesthesia involving feet and hands was added. Neurological examination showed diminished power in the four limbs and generalized areflexia. Treatment was administered identically to the previous case. The patient showed great improvement until complete recovery.

Another review by Sriwastava et al. (2021) analyzed 50 GBS-affected patients with concomitant molecularly confirmed SARS-CoV-2 infection, by layering them depending on GBS variants. Notably, 33 patients had the AIDP phenotype and the remaining 17 had other variants. The authors did not include pediatric cases. Of the total 50 reviewed patients, 30 had severe COVID-19 requiring mechanical intubation. Of all patients, 44 received IVIG; of the latter, 30 patients (68%) had AIDP, while 14 (32%) did not. The authors distinguished patients receiving IVIg 0.4 g/kg/day and 2 g/kg, both over 5 days. Of the 14 cases (10 in the AIDP group and 4 in the non-AIDP group), information about different IVIG regimens was not available. A total of 14 patients (70%) in the AIDP group received 0.4 g/kg/day and the other 6 patients (30%) received 2 g/kg IVIg regimen, all within 5 days. In the non-AIDP group, the total patients on 0.4 g/kg and 2 g/kg IVIg regimens were 8 (80%) and 2 (20%) respectively. In total, out of 30 patients on IVIg in the entire cohort, 22 patients (73%) were on 0.4 g/kg dosage and 8 (27%) were on 2 g/kg dosage divided over 5 days. Most patients had either full recovery or partial recovery, whereas five of them died. No adverse events were reported.

In a multicenter observational study by Filosto et al. (2021), a total of 25 patients affected by COVID-19-related GBS were treated with IVIg. Males were the 73% of IVIg + non-IVIg-treated patients, while the mean age of them was of 59.2 ± 12.1 years. The bulk of patients were treated with the standard IVIg regimen of 0.4 g/kg/day for 5 days. A clinical improvement was reported in at least 80% of cases. One more time, no data about side effects were noticed. In the same study, data concerning a group of 17 patients with GBS but without SARS-CoV-2 infection were also collected. Of those, 16 were treated with IVIg. Most importantly, according to the authors, the response to therapy was not different in COVID-19-positive and COVID-19-negative patients. While this fact strengthens the evidence that the therapeutic scheme currently in use is effective, it does not justify the dose changes in the two concomitant conditions.

Two other clinical studies performed at the beginning of the pandemic by Manganotti et al. (2020, 2021a) move in the same direction. A case series of four males aged 72, 72, 76, and 94 years and a 49-year-old female were examined. They presented with various neurological symptoms of a COVID-19-associated GBS spectrum, such as limb weakness up to flaccid tetraparesis, absence of deep tendon reflexes, and eventual cranial nerve involvement. The last patient clinically presented with the symptomatologic triad of MFS, which consists of ophthalmoplegia, ataxia, and areflexia. After a single cycle of IVIg at the dosage of 0.4 g/kg/day for 5 days, all subjects experienced a partial resolution of symptoms without developing adverse effects.

Another rare case of COVID-19-related cranial polyneuritis in the framework of a GBS was described by Khaja et al. (2020). A 44-year-old man complained about a bilateral facial weakness, with the inability to raise eyebrows, close eyes, smile, and feel taste. The patient did not have dysarthria or dysphagia. He had normal limb strength and deep tendon reflexes as well as oculomotor movements and gait. A severe bilateral dysfunction of the facial nerve was observed. There was no hyperacusis and the corneal reflex was intact. All other cranial nerves were undamaged. A nasopharyngeal molecular swab was performed, resulting in positive. CSF analysis showed typical albuminocytologic dissociation with elevated protein levels (92 mg/dL) and no leucocytes. A magnetic resonance imaging of the cervical spine did not show any abnormal enhancements. No IgG antibodies against GQ1B were detected in serum. A classical cycle of 0.4 g/kg/day of IVIg for 5 days was administered. On day 10 from admission, the patient was able to close both eyes and on day 12, he was discharged. Adverse effects were not reported.

Assini et al. (2020) reported other two cases of COVID-19-related GBS spectrum with significant impact on cranial nerves and gastrointestinal functions, respectively. Both presented with a severe respiratory syndrome requiring hospitalization. The first one was a 55-year-old man who after 20 days from admission developed acute onset of bilateral eyelid ptosis, dysphagia, dysphonia, bilateral paralysis of the hypoglossal nerve, and hyporeflexia of upper and lower limbs. Electroneurography revealed symmetrical demyelination and typical sural sparing patterns in four limbs. Diagnosis of overlapping GBS/MFS was formulated. The second was a 60-year-old patient who after 20 days from admission presented with gastroplegia and paralytic ileus, acute weakness in lower limbs with distal distribution, as well as right foot drop and the absence of deep tendon reflexes. Electroneurography showed severe sensory-motor axonal polyneuropathy and electromyography showed neurogenic changes in the limbs. A diagnosis of AMSAN was made. Both cases were treated with a standard cycle of IVIg at the dosage of 0.4 g/kg/day for 5 days. The first patient showed first clinical improvements during the fifth day of treatment, with a rapid improvement of all symptoms and a gradual and complete recovery of swallowing and feeding. The second one showed clinical improvements after 5 days—a substantial remission of vegetative symptoms and a slight improvement of the right foot drop, with persistent hyporeflexia. In both cases, no adverse events of therapy were observed.

Gutiérrez-Ortiz et al. (2020) described the case of a 50-year-old man who presented with flu-like symptoms, followed after 5 days by anosmia, ageusia, right internuclear ophthalmoparesis, right fascicular oculomotor palsy, ataxia, and limb areflexia, leading to the diagnosis of MFS. Laboratory findings included, other than positivity for SARS-CoV-2 infection, a liquoral albuminocytologic dissociation and a positive testing for anti-GD1b–immunoglobulin G antibody. The patient was treated with a typical cycle of IVIg of 5 days at the dosage of 0.4 g/kg/day. Two weeks later, he obtained complete neurological recovery, except for residual anosmia and ageusia. No side effects of therapy were reported.

Garcia et al. (2021) elucidated the efficacy and safety of IVIg administration even in the particular condition of COVID-19-related GBS during pregnancy. They reported the case of a 22-year-old gravida at 20 weeks age of gestation presenting with fever and respiratory/gastrointestinal symptoms. A week after they were followed by acroparaesthesias, limb weakness, dysphonia, and dysphagia. Her medical, family, and personal-social history were silent for pathologies. Lumbar puncture did not show albuminocytological dissociation. Nerve conduction studies were initially delayed but would later confirm the diagnostic suspicions. In view of the clinical picture, a 5-day course of intravenous immunoglobulin (IVIg) at the dosage of 0.4 g/kg/day was engaged. Because of the therapy, the patient reported improvements in dyspnea and weakness, without any side effects. This last fact is particularly significant because both pregnancy and COVID-19 are prothrombotic conditions (D'Souza et al., 2020), while IVIg therapy is commonly regarded as such. Yet, Sakoulas et al. (2020) reported that it can even diminish the risk of coagulopathy by reducing vascular inflammation. On day 13 post-IVIg, the patient was discharged and within 1 month bulbar and motor symptoms were substantially resolved. Pregnancy finally ended with vaginal birth.

Scheidl et al. (2020) described the case of a 54-year-old woman hospitalized with acute, mainly proximal, symmetrical paraparesis. Other symptoms were areflexia, numbness, and tingling of all extremities. These began 3 weeks after a noticed positivity for SARS-CoV-2 infection and were already progressing for 10 days from admission. She experienced only a transient loss of smell and taste 2 weeks before the symptoms of GBS occurred. CSF examination showed typical albuminocytologic dissociation with increased protein level (140 g/L) and normal cell count. Electromyography showed no denervation signs. Electrophysiological findings were consistent with AIDP. A worsening of paraparesis and the emergence of dysphagia were observed 2 days after admission. Therefore, the patient was treated with IVIg at a dosage of 0.4 g/kg/day for 5 days after which the patient reached almost complete recovery. No side effects of therapy were mentioned.

Reyes-Bueno et al. (2020) described a case of COVID-19-related MFS concerning a 51-year-old woman, who had presented with the onset of flu-like symptoms 2 weeks before. She started having intense root-type pain in all four limbs, especially in the legs and lower back. Then she developed leg weakness until walking was impossible and double binocular vision, leading to hospitalization. The neurological examination highlighted a left external rectus muscle paresis with horizontal diplopia, bilateral facial paresis, symmetrical paraparesis, and global areflexia. Autonomic dysfunction was also present. An albumin-cytological dissociation in CSF was found. Antiganglioside antibodies were negative. Neuroimaging studies and the other laboratory analyses were negative. Neurophysiological findings were compatible with demyelinating polyneuropathy. The patient was treated with IVIg at the classical dosage of 0.4 mg/kg/day for 5 days and gabapentin 900 mg/day. The patient showed progressive improvement in facial and limb paresis, diplopia, and pain, without any side effects of therapy.

Another case of probable COVID-19-related MFS was reported by Dinkin et al. (2020). It concerned a 36-year-old man who presented with left ptosis, diplopia, and distal paraesthesia on both legs, preceded by flu-like symptoms. The neurological examination highlighted a left oculomotor palsy with the involvement of the intrinsic component. Bilateral abducens palsy was observed, too. Ocular manifestations were accompanied by lower extremity hyporeflexia, hyperesthesia, and gait ataxia. MRI revealed an inflammatory process affecting the left oculomotor nerve. The patient received IVIg at the dosage of 2 g/kg for 3 days and showed partial improvement of deficits. No adverse events were registered.

### 5.2. Encephalitis/meningoencephalitis

Yousuf et al. (2021) described the case of a 60-year-old male who recovered from COVID-19 9.5 weeks prior to admission to the neurology department. He presented with a plethora of new neuropsychiatric symptoms including hallucinations, perseverations, impulsive behaviors, episodes of gaze deviation and unresponsiveness, word-finding difficulties, fluctuating memory, cognitive deficits, child-like regression, aphasia, and paranoia. After a brief hospital stay of 4 days, he was discharged home. But 2 days later familiars, due to a new worsening, brought him back. On neurological examination, the patient showed akinetic mutism and bradykinesia, as well as the aforementioned disturbances in reasoning. After treatment with the classical IVIg regimen of 0.4 g/kg/day for 5 days, his symptoms strongly improved as verified by successive neuro-cognitive testing. No side effects of therapy were reported.

Chenna et al. (2021) illustrated the case of a 58-year-old who after a SARS-CoV-2 infection developed altered consciousness, agitation, and confusion, without focal neurological deficits. Deep tendon reflexes were absent. In view of the absence of improvements and in the suspicion of an ADEM, a typical cycle with IVIg at the dosage of 0.4 g/kg/day for 5 days was undertaken. The patient fully recovered already on day 3 after the start of treatment. After 1 month, the patient was able to walk independently and no side effects were reported.

McAlpine et al. (2021) showed the case of a 30-year-old man who after a SARS-CoV-2 infection began to experience severe hypersomnia and subsequently, insomnia, and overt psychosis. He was successfully treated with haloperidol and discharged after 48 h. After discharge, insomnia and cognitive disturbances recurred. On neurological examination apathy, slowed speech, and akathisia were observed. Neuroimaging and video-electroencephalography were negative. In CSF, an elevated amount of IgG was detected. At that stage, the patient received a total amount of 2 g/kg of IVIg over 3 days, which resulted in a drastic improvement in his cognitive and psychotic symptoms and sleep cycle disorders. He was discharged, immediately returned to work, and maintained symptom freedom for the following 3 months. No adverse effects of therapy were mentioned.

Another work by Burr et al. (2021) was focused on the case of a 23-month-old toddler who in the progress of COVID-19 appeared non-interacting, non-talking, and most frequently suffered from shaking arms and legs. Two days after hospital admission, she had several seizures and was acutely treated with lorazepam and levetiracetam. All CSF analyses came negative. Brain magnetic resonance was normal. Even after the resolution of fever, the patient continued to have symptoms of encephalopathy and lingering hyperkinetic movements of extremities. Intravenous methylprednisolone was administered for 5 days without effects. Positivity for IgG autoantibodies directed against N-methyl-D-aspartate (NMDA)-receptors both in serum and CSF. All microbiological tests except those for SARS-CoV-2 were negative. Due to the persistence of symptoms, IVIg at the dosage of 2 g/kg was administered (no information was provided for the time duration of the therapy), with gradual resolution over the next week. No side effects of therapy were reported.

In a case report by Gaughan et al. (2021) a 16-year-old female presenting with a picture of COVID-19-associated encephalitis, with severe neuropsychiatric symptoms such as akinetic mutism, hallucinations, bilateral limb rigidity, and tremor. She was treated with one cycle of the classical IVIg regimen of 0.4 g/kg daily for 5 days. No side effects were collected in that circumstance. A second cycle of IVIg was started, but it was interrupted due to the appearance of a widespread rash, a possible adverse cutaneous reaction. Nevertheless, the patient experienced a nearly complete recovery after 6 months.

Fukushima et al. (2021) exposed the case of a 20-year-old previously healthy man who was admitted because of a persistent headache. Six weeks prior to this admission, he was hospitalized for a new onset seizure, confirmed by electroencephalography. The MRI of the brain revealed hyperintensity of the left frontal gyrus. The patient was therefore discharged with an antiepileptic therapy. Four weeks after, he had another seizure and developed a throbbing headache over the left frontal region, nausea, and emesis. Thus, he was hospitalized for the second time. The neurological examination proved unremarkable. CSF samples showed lymphocytic pleocytosis, raised protein levels, and elevated IgG amount. The cranial magnetic resonance imaging showed increased signals over a wider area than that of the previous exam. Serum IgG for SARS-CoV-2 was positive. IVIg at the typical dosage of 0.4 g/kg/day for 5 days was administered. The headache completely resolved and after 2 months the patient was symptom free, an observation also supported by imaging negativity.

Another clinical study by El-Zein et al. (2020) considered a 40-year-old male with COVID-19-associated meningoencephalitis. In the neurological assessment, manifestations included altered mental status, self-centered orientation, and inability to execute orders. He was subjected to one cycle of the standard IVIg treatment of 0.4 g/kg/day for 5 days. Soon after the IVIg treatment, thanks to a drastic clinical improvement, he was dismissed without reported adverse events. Two months later, all symptoms had disappeared.

Nevertheless, IVIg is revealed to be effective even against COVID-19-associated ADEM, according to the results of a clinical study by Parsons et al. (2020). The present case concerned a 51-year-old female without past neurological history who developed severe unresponsiveness up to coma, flaccid muscular tone, and generalized hyporeflexia. In the accompaniment, she presented with multiple cerebral lesions in MRI sequences and xanthochromism liquor upon lumbar puncture. After a typical cycle of IVIg at the dosage of 0.4 g/kg/day for 5 days, alertness improved gradually and the patient recovered the ability to execute elementary orders, without developing adverse effects.

### 5.3. Encephalopathy

A case report by Delamarre et al. (2020) focused on the picture of a 51-year-old man suffering from acute necrotizing encephalopathy, perhaps mediated by antibodies. His individual and familial history was negative for any neurological disorder. He contracted and was treated for COVID-19. After oxygen weaning, he dramatically developed progressive unresponsiveness up to coma with altered blood and cerebrospinal samples, likewise multiple pathological MRI and electroencephalographic findings. He was treated with a total dose of 2 g/kg of immunoglobulins over 5 days. In the space of some days after the end of the cycle, he experienced complete motor recovery, ameliorated cognitive dysfunction, and significant improvement of lesions previously evident on MRI. In addition, IVIg therapy turned out to be devoid of side effects.

Muccioli et al. (2021) explored the cases of five patients affected by COVID-19-related encephalopathy, too. They were three males and two females with a mean age of 66.8 years. Their neurological status was variously represented by disturbances of consciousness, pyramidal and extrapyramidal signs, eventual frontal release reflexes, and psychiatric manifestations. They were submitted to an IVIg dosage of 0.4 g/kg/day for a minimum of 3 days and a maximum of 5 days. An almost complete clinical, electroneurographic, and radiological recovery was obtained soon after the therapy, and none of the patients experienced any kind of adverse events. Neurological negativity was reached upon successive follow-up visits.

Abdel-Mannan et al. (2020) described the case of a 15-year-old female with COVID-19-related encephalopathy. Her neurological symptoms consisted of confusion, disorientation, headache, global proximal weakness, and reduced deep tendon reflexes. Lumbar puncture and electroencephalography were not performed. Magnetic resonance imaging showed signal changes in the splenium of the corpus callosum with mildly restricted diffusion. The patient was treated with a single dose of 1 g/kg of IVIg. Encephalopathy resolved, the patient regained normal ambulation, and was discharged after 18 days. No adverse effects of therapy were mentioned.

Delorme et al. (2020) reported the case of a 72-year-old man, who 15 days after COVID-19 onset, presented with acute psychomotor agitation, cognitive and behavioral frontal lobe symptoms, upper limb myoclonus, and cerebellar ataxia. A CSF examination revealed 6 cells/mm^3^. Brain MRI resulted unremarkable. Brain F-18 deoxyglucose (FDG) positron emission tomography/computed tomography (PET/CT) imaging 23 days after COVID-19 onset showed bilateral prefrontal and left-sided parietal-temporal hypometabolism and slight hypermetabolism within the cerebellar vermis. Owing to the suspicion of encephalitis, IVIg was administered at the dosage of 2 g/kg (no information was provided for the time duration of the therapy), without reported side effects. All the aforementioned symptoms improved gradually after IVIg, even with the resolution of cognitive dysfunctions 6 weeks after COVID-19 onset.

### 5.4. New-onset refractory status epilepticus

IVIg therapeutic potential against COVID-related refractory status epilepticus of new-onset (NORSE) has been successfully investigated by a clinical study by Manganotti et al. (2021b) Two males aged 37 and 71 years were analyzed. Upon electroencephalography, both presented with generalized epileptic discharges compatible with non-convulsive status epilepticus. The diagnosis of a possible autoimmune encephalitis-related NORSE (seropositive in the first case and seronegative in the second) was done based on laboratory findings together with the lack of response to multiple antiepileptic drugs and third-line anesthetic drugs. The IVIg regimen consisted of 0.4 g/kg/day for 5 days for one cycle in the first case and two cycles in the second. This therapy resulted in a partial decrease in antiepileptic drugs without severe residual cognitive impairment. No side effects were highlighted.

Leelamani and Mohammed Rajab (2021) reported the case of a young adult who developed multiple COVID-19-related tonic posturing seizures with uprolling of eyes. Diazepam, phenytoin, dexamethasone, and empirical antimicrobial therapy based on ceftriaxone and acyclovir for meningitis were administered. However, the patient continued to have seizures. Thus, she was intubated, mechanically ventilated, and the intravenous antiepileptic therapy was upgraded. CSF analysis and brain CT were normal. Due to the lack of tangible effects, first phenobarbital and then propofol, thiopentone, and ketamine were tried, although without benefits. Finally, a cycle of IVIg at the typical dosage of 0.4 g/kg/day was engaged. After 2 days, no more seizures were observed, and it was possible to progressively stop anesthetic medications. The recovery of consciousness, eye opening, and motor reaction to painful stimuli in the four limbs were achieved after 5 days. No further seizures occurred. No side effects were reported. This demonstrates how IVIg therapy might be decisive in treating NORSE even in the case of failure of all other suitable drugs.

### 5.5. Neuropathy/dysautonomia

Stoian et al. (2021) elucidated for the first time the therapeutic role of IVIg in COVID-19-related critical illness polyneuropathy. The authors dealt with the case of a 54-year-old woman affected by severe pneumonia due to COVID-19, requiring admission to the intensive care unit. Since then, she developed increasing motor deficit first in the lower and later in the upper limbs and up to flaccid tetraparesis. Reduced and no deep tendon reflexes were observed in the upper and lower limbs, respectively, along with gloves-and-socks hypoesthesia and hypopallesthesia. The level of creatine kinase was normal during the stay. Lumbar puncture and MRI of the brain and spine were unremarkable. Nerve conduction studies were consistent with axonal loss, while the electromyography demonstrated a pattern of ongoing denervation in the distal muscles of the lower limbs. The patient received an IVIg treatment at the dosage of 0.4 g/kg/day for 5 days, without reported side effects. After 16 days, she exhibited a visible recovery of respiratory functions and strength, up to nearly complete in the following months.

In a single case report by Novak (2020), a 64-year-old female with a previous history of Lyme's disease, headaches in hypothyroidism, painful small fiber neuropathy, and orthostatic hypoperfusion syndrome. Some typical features of her pathologies, such as headaches, distal burning pain sensation, blurred vision, brain fog, and chronic fatigue, especially in orthostatic position, appeared even with more emphasis after the onset of the COVID-19 respiratory symptoms. Autoimmune mechanisms were suspected as responsible for this clinical worsening. Thus, she received an IVIg treatment of 2 g/kg/month for 2 months, which was then decreased to 1 g/kg/month due to concomitant headaches. A marked improvement of all symptoms was obtained, without recorded adverse reactions to the lower dosage.

Another case report by Saleh et al. (2021) focused on a 56-year-old woman presenting with painful dysesthesia in the extremities after COVID-19. Over a few days, the patient developed a general weakness with walking disturbances. Upon neurological examination, a positive Phalen test, exaggerated patellar and deep tendon reflexes of the upper limbs, weak Achilles reflexes, and distal symmetrical hypoesthesia and hypopallesthesia were observed. Electrophysiological findings were consistent with acute/chronic inflammatory demyelinating polyneuropathy. Thickened nerve roots were demonstrated via ultrasound. Autonomic testing showed the involvement of the relative system. Spinal fluid analysis displayed a discrete albuminocytological dissociation. An MRI of the cervical column did not reveal spinal cord affections. A classical IVIg regimen of 0.4 g/kg/day for 5 days was carried out, with marked motor, sensory, and electrophysiological picture improvement and without any complications except for a slight headache. Even a second dose of 90 g of IVIg was administered after 4 weeks, without the appearance of side effects.

### 5.6. Myositis

Gupta et al. (2021) reported the case of a 49-year-old female patient presenting with a subacute symmetrical proximal muscle weakness of all four limbs, characterized by difficulty in getting up, lifting objects, and walking without support. It was accompanied by severe myalgia involving both arms and thighs. In the neurological examination, no muscle atrophy and normal reflexes, sensitivity, and cranial nerves were objectified. The only significant precedent was a recent silent SARS-CoV-2 infection. Magnetic resonance imaging and laboratory findings were consistent with myositis. The patient received 2 g/kg IVIg divided over 5 days without any reported side effects. She showed rapid improvement of symptoms within 24 h, with the termination of myalgias and tenderness and full recovery of motor power in the four limbs in a few weeks.

### 5.7. Myasthenic crisis

IVIg therapy is a well-consolidated treatment for myasthenia gravis exacerbations (Gajdos et al., 2012). This effectiveness was confirmed even in a patient with myasthenic crisis and concomitant COVID-19 syndrome in a case report by Delly et al. (2020). A 56-year-old female with diffuse myalgias and deteriorating respiratory function up to requiring mechanical ventilation was evaluated. She received her first cycle of IVIg at the typical dosage of 0.4 g/kg/day for 5 days and a second cycle of 650 mg/kg for 2 days in a row. After the first cycle, an important improvement of the respiratory distress was achieved; after the second, proximal limb weakness was ameliorated and the patient recovered the ability to stand up. No side effects were observed.

Furthermore, Huber et al. (2020) reported a rare case of the onset of myasthenia gravis after a SARS-CoV-2 infection. A 21-year-old woman presented with double vision and right-sided ptosis within 3 weeks after COVID-19. The patient's medical history was noteworthy only for familiarity with non-muscular autoimmune disease. A neuro-ophthalmological examination showed a right eye elevation deficit. An MRI with a contrast of the brain and orbits was normal. Electrophysiological examinations were no diriment. An intravenous edrophonium chloride test resulted positive. The antibodies against acetylcholine receptors were elevated in serum. The CSF was positive for oligoclonal bands. Symptoms of dysphagia and dyspnoea lacked. The patient was treated with oral pyridostigmine and IVIg at the classical dosage of 0.4 g/kg/day for 5 days, obtaining a regression of ocular disturbances. No side effects were reported.

### 5.8. Opsoclonus myoclonus syndrome

Ishaq et al. (2021) discussed the case of a 63-year-old man who, 23 days after the onset of COVID-19, developed neuropsychiatric symptoms and above all involuntary saccadic multidirectional eye movements and myoclonic jerks of the limbs. CSF analysis was normal, as well as the MRI of the brain. A diagnosis of opsoclonus myoclonus was posed. Malignancies or paraneoplastic conditions were excluded. Screening tests for autoimmune encephalitis resulted negative. The patient was treated with methylprednisolone 1 g/day for 5 days, but without beneficial effects. Subsequently, IVIg was administered at a dosage of 2 g/kg in five divided intakes. The patient exhibited a gradual complete recovery over the next 4 weeks, without developing side effects. Interestingly, this case demonstrates how steroid-unresponsive patients may have a positive reaction to IVIg therapy.

## 6. Summary of side effects of IVIg

According to a review by Guo et al. (2018), the most frequent side effects of IVIg are a flu-like syndrome (about 80% of patients), headache (about 50% of patients) and, far less, dermatological reactions (about 6% of patients). These symptoms are mostly mild to moderate and self-limiting.

Severe adverse events, such as heart or brain infarctions, renal failure, and cardiac arrhythmias are infrequent and their underlining mechanisms are not completely recognized. As for thrombosis concerns, it might be linked to IVIg-induced plasma hyperviscosity, although the coexistence of other risk factors and a previous history of vascular events has been identified as being strongly associated in the majority of cases. To a certain extent, all side effects of IVIg appear to be diminished by reducing the intravenous infusion velocity, especially at the beginning of treatment for the most common.

Regarding the contraindications, the presence of minor components in IVIg formulations may constitute a caveat, such as glucose for diabetics, sucrose for kidney disease, and high IgA concentrations for subjects prone to allergic reactions (Lemm, 2002).

Of all the 32 studies analyzed in this review, we have noticed that the majority, i.e., 28 studies, did not report side effects for IVIg; of these, six did not contain any data in this regard. A total of three studies reported adverse reactions, mild in all cases: a widespread rash during a second cycle of IVIg (no side effects due to the previous cycle) and two cases of IVIg-related headaches, one of which only after the first of two cycles and not present after the second in the same subject. Finally, one study denied severe side effects (no information about minor ones).

Although some studies did not report data about side effects, the picture so far represented seems to delineate a large overall safety profile of IVIg employment to treat COVID-related neurological conditions.

## 7. Discussion and closing remarks

Despite at the time of this review more than 2 years have passed since the first case of COVID-19, it can be reasonably considered a relatively short time during which the scientific community has put great efforts to understand the pathophysiological mechanisms of the infection and to develop and study potential therapeutic options to treat often severe conditions. This was particularly evident when, during the 1^st^ months of the pandemic, many patients presented a variety of symptoms and complications, and there was a lack of commonly accepted therapeutic procedures. The present review represents an up-to-date report of the main findings about the use of IVIg therapy in COVID-19 neurological conditions, which can still be present, and therefore can represent a useful inspiration for clinical practice. First, IVIg is a versatile therapeutic tool with multiple molecular targets and mechanisms of action reflecting the complexity of the structures and functions of human antibodies, as confirmed by the long list of IVIg therapeutic indications (Arumugham and Rayi, 2022). In these 2 years of the SARS-CoV-2 pandemic, several neurological complications have been extensively reported. The epidemiology and the mechanisms of neurological complications are still controversial and under investigation. For instance, the CNS manifestations are far from the classical autoimmune encephalitis picture associated with known neuronal antibodies. However, in clinical practice, the impact of IVIg treatment both on inflammatory and autoimmune processes in numerous neurological complications of COVID-19 is testified by a considerable number of successful reports, as illustrated in this narrative review. By analyzing the cases dealt with so far, we have been able to ascertain how IVIg effects are fairly quick to manifest themselves (from hours to days for the onset) and long-lasting over time (in the order of weeks or months), when not decisive (Van den Berg et al., 1998). Dosages, outcomes, and side effects, which seldom occurred, were entirely consistent with the usual ones in clinical practice (Willison et al., 2016; Uncini et al., 2020).

In GBS/MFS, clinical presentation and response to the overall treatment do not indicate a clear distinct entity triggered by SARS-CoV-2, but from a pragmatic point of view IVIg was however a successful therapy against all the peripheral neurological pathologies in patients affected by SARS-CoV-2. For all the other severe neurological conditions with an unfavorable prognosis, such as encephalitis, myelitis, and ADEM, IVIg was reported to be beneficial in several reports during 2 years of the pandemic. Even in the case of multidrug resistance, like the NORSE cases, a significant response was obtained, indicating its superiority over symptomatic disease-specific drugs and suggesting the complex pathways of COVID-19-induced encephalitis. In most treated cases, IVIg therapy did not present adverse reactions and has been proven safe during pregnancy (Han et al., 2021). Considering the possibility of side effects consequent to the administration of high-dose corticosteroids (Yasir et al., 2022), IVIg represents a valid and safe alternative to other common therapies. For all the above reasons, besides the well elucidated and not negligible role in the SARS-CoV-2-induced constellation of non-neurological conditions (Gharebaghi et al., 2020; Mohtadi et al., 2020), IVIg treatment should be carefully considered as a potent therapeutic weapon against several neurological manifestations associated with COVID-19 syndrome, particularly in critically ill patients and those unresponsive to other medications.

Although empirical immunotherapy is beneficial for some COVID-19 patients with neurological complications, a better understanding of the pathophysiology to determine the subset of cases that is more likely to benefit from immunotherapy, if immunotherapy can prevent neuropsychiatric post-acute COVID-19 sequelae and is the best therapeutic approach required. The diagnosis is also challenging, as in cases with CNS involvement the criteria even for possible autoimmune encephalitis were lacking in 26% (20/76) of this aggregated series and the criteria are definite only in 16% (12/76, only two without antibodies).

This highlights the need for novel biomarkers, and further studies should explore in a more systematic way the utility of neuroimaging, CSF profiles including cytokines, and search for hitherto-unknown neuronal antibodies. Finally, for those patients with pre-existing neuroimmunological disorders treated with immunotherapy, the accumulated evidence has raised no safety concerns so far, but caution should prevail in the decision-making process.

## Author contributions

PM made substantial contributions to the conception and substantively revised the work. AB, GG, and PM made the design of the work. GG, AB, and GF made the acquisition, analyses, interpretation of data, and have drafted the work. All authors have approved the submitted version of the work and have agreed both to be personally accountable for the author's own contributions and to ensure that questions related to the accuracy or integrity of any part of the work are appropriately investigated, resolved, and the resolution documented in the literature.
